# Electroacupuncture Attenuated Anxiety and Depression-Like Behavior via Inhibition of Hippocampal Inflammatory Response and Metabolic Disorders in TNBS-Induced IBD Rats

**DOI:** 10.1155/2022/8295580

**Published:** 2022-01-18

**Authors:** Feini Zhou, Hao Jiang, Ning Kong, Jiangnan Lin, Fan Zhang, Ting Mai, Zhijian Cao, Maosheng Xu

**Affiliations:** ^1^The First School of Clinical Medicine of Zhejiang Chinese Medical University, Hangzhou, China; ^2^Department of Radiology, The First Affiliated Hospital of Zhejiang Chinese Medical University, Hangzhou, China

## Abstract

This study was designed to explore the potential mechanisms of electroacupuncture (EA) in treating inflammatory bowel disease- (IBD-) related anxiety and mood disorders. A colitis model was induced in rats with 2, 4, 6-trinitrohydrosulfonic acid (TNBS), followed by ST36 and SP6 targeted therapy by EA or sham EA treatment. The elevated plus maze (EPM) and open-field test (OFT) were performed to assess the state of anxiety and depression-like behavior. Tests were carried out by 16S rDNA amplification sequence, ^1^H nuclear magnetic resonance (^1^H NMR) spectroscopy, immunofluorescence staining, and enzyme-linked immunosorbent assay (ELISA). The analyses detailed metabolic alterations and the Toll-like receptor 4 (TLR4) signaling pathway/NOD-like receptor protein 3 (NLRP3) inflammasome in rats' hippocampal region. Furthermore, the activity of the hypothalamic-pituitary adrenal (HPA) axis and gut microbiome was assessed. As a result of treatment, EA significantly improved in the behavioral tests and altered the composition of the gut microbiome through a significant increase in the density of short chain fatty acids (SCFAs) producers mainly including *Ruminococcaceae*, *Phascolarctobacterium*, and *Akkermansiaceae*. EA upregulated the metabolites of the hippocampus mainly containing l-glutamine and gamma-aminobutyric acid (GABA), as well as ZO-1 expression. Whereas the treatment blocked the TLR4/nuclear factor- kappa B (NF-*κ*B) signaling pathways and NLRP3 inflammasomes, along with downregulating the interleukin- (IL-) 1*β* level. The hyperactivity of the HPA axis was also diminished. In conclusion, EA at ST36 and SP6 attenuated anxiety and depression-like behavior in colitis model rats through their effects on the gut microbiome by modulating the hippocampal inflammatory response and metabolic disorders, as well as the HPA axis. This study provides evidence for clinical application of EA to serve as an adjunctive treatment for IBD-related anxiety and depression.

## 1. Introduction

Inflammatory bowel disease (IBD) is a group of disorders including Crohn's disease (CD) and ulcerative colitis (UC) [1]. Both are chronic, immunological disorders with increasing incidence [2]. IBD is associated with depression [3, 4]. The incidences of psychiatric disorders are significantly higher for IBD patients when compared to the general population. A previous study reported that the prevalence of anxiety and depressive disorders in IBD patients reached 25.8% and 21.2%, respectively [5]. Recently, a study considered the prevalence to be as high as 33.1% [6].

It has been proved that anxiety and mood disorders would exacerbate IBD activity and decrease the quality of life of patients [4, 5]. Therefore, the psychophysiological vulnerability of IBD patients highlights the importance of treating their psychological requirements [7].

For traditional antidepressants, therapeutic effects can take weeks to effect [8], which would be outside the therapeutic window for most IBD patients in the acute phase. Electroacupuncture (EA) is a modern technique derived from traditional Chinese therapeutic acupuncture. The mechanism is based on the philosophy that currents spread to nearby areas and can affect peripheral nerve impulses (action potentials) more intensely [9]. When used in conjunction with medical therapy, the rapid onset of therapeutic effects could augment the antidepressant effect [10–12]. In addition, EA has anti-inflammatory effects, promotes the restoration of intestinal microecology to homeostasis, and helps to maintain mucosal tight junctions [13, 14]. Therefore, it was speculated that EA may be a promising procedure for IBD-related anxiety and depression.

Central nervous system (CNS) inflammation is associated with anxiety and mood disorders. The characteristic inflammatory response evokes the sustained activation of microglia and astrocytes and the release of inflammatory cytokines which contributes to the development of psychiatric disorders such as anxiety and depression [15, 16]. The hippocampus plays a critical function in learning and memory and could be a key brain region involved in depression [17] as alterations of hippocampal metabolomics have been discovered in depression model rats [18]. And the hypothalamic-pituitary adrenal (HPA) axis is associated with the development of anxiety, major depressive disorder, and other stress-related conditions [19]. The hippocampus showed high levels of corticosteroid receptors (GRs) which interact with corticosterone (CORT) to trigger signaling pathway and metabolic changes in the hippocampal region during the stress response [20, 21]. Moreover, through the dynamic bidirectional communication systems between the immune, metabolic, and nervous systems of the microbiome-gut-brain (MGB) axis, anxiety and depression disorders may affect alterations in gut microbiota diversity, community composition, and vice versa [22, 23]. Our previous study has supported that the disturbance of MGB axis was associated with depression-like behavior in rats [24]. Therefore, this study seeks to examine the potential mechanisms of EA treatment on the relief of IBD-related anxiety and depression.

We performed EA intervention at the acupuncture points of ST36 and SP6 on 2, 4, 6-trinitrohydrosulfonic acid- (TNBS-) induced colitis model rats. It was found that ST36 ameliorates inflammation and increases the expression of tight junction proteins [25, 26] and SP6 can relieve pain [27]. In combination, both of them can affect the HPA axis [28]. Subsequently, we conducted behavioral tests and assessed the gut microbiota by 16S rDNA amplification sequencing, detected the alteration of metabolites in the hippocampus by ^1^H nuclear magnetic resonance (^1^H NMR), examined the Toll-like receptor 4 (TLR4) signaling pathway/NOD-like receptor protein 3 (NLRP3) inflammasome in the hippocampal region by immunofluorescence staining, and analyzed the activity of HPA axis by enzyme-linked immunosorbent assay (ELISA). The overlap between depressive and anxiety symptoms was also observed [29].

## 2. Materials and Methods

### 2.1. Animals, Experimental Design, and Sample Collection

Experimental rats (male, 8-weeks, Sprague Dawley) were purchased from Shanghai B&K Co., Ltd. (Shanghai, China). All rats were housed in the Laboratory Animal Center of Zhejiang Chinese Medical University at 21 ± 2°C and 55 ± 10% humidity with free access to autoclaved chow and water. After acclimation to the environment for 7 days, the animals were randomly divided into 3 groups (*n* = 8/group): control, TNBS, and TNBS + EA group. Rats fasted for 24 h (water ad libitum) and anesthesia with 3% pentobarbital was administered. TNBS solution (50 mg/kg; Sigma-Aldrich, Steinheim, Germany) dissolved in ethanol (50% *v*/*v*) was instilled at a dose of 1 mL/kg rat body weight for inducing the colitis model [30]. A 3 mm diameter rubber catheter was inserted 8 cm proximal to the anus. The dose was slowly administered, and the catheter was clamped in the anus for one minute to prevent solution leakage.

**Figure 1:**
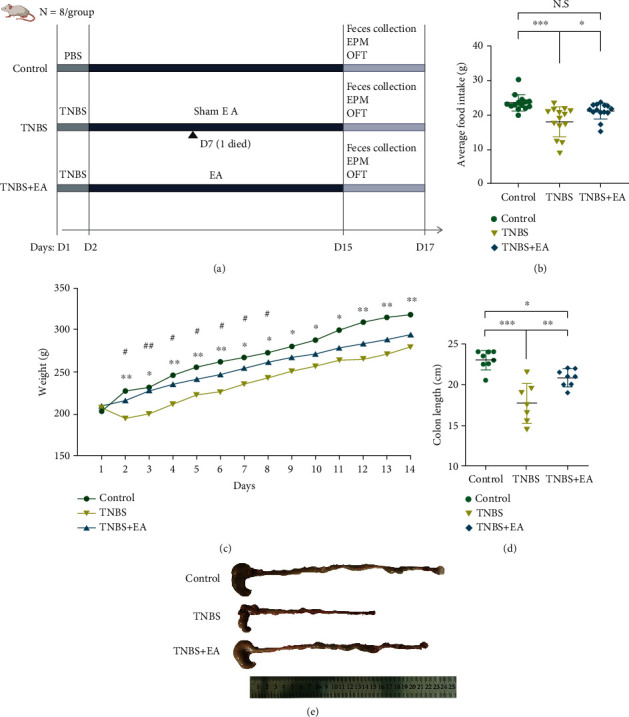
Animals (control (*n* = 8), TNBS (*n* = 7), and TNBS + EA group (*n* = 8)). (a) The experimental process. (b) The average food intake of each rat was calculated. (c) Body weight was recorded. ∗, ∗∗: control vs. TNBS group; ^#^, ^##^: TNBS vs. TNBS + EA group. (d) and (e) The colon length was measured. Data were shown as mean ± SD. ∗*P* < 0.05, ∗∗*P* < 0.01, ∗∗∗*P* < 0.001 by Bonferroni's post hoc test or independent sample *t* test, and ^#^*P* < 0.05, ^##^*P* < 0.01 by independent sample *t* test.

Symptoms of diarrhea, hematochezia, and weight loss were apparent on the first day following treatment. The control group received phosphate buffer saline (PBS (1.9 mM Na2HPO4, 8.1 mM NaH2PO4, 150 mM NaCl, pH 7.4)) instead of TNBS solution. ST36 (Zusanli, 4-5 mm lateral to the anterior tibial tuberosity) and SP6 (Sanyinjiao, 3 mm proximal to the medial malleolus at the posterior border of the tibia) were selected as the acupuncture point and inserted bilaterally to a depth of approximately 7 mm, 5 mm after disinfection, respectively [28]. The parameters were implemented with 5 Hz and 0.2 mA (Hwato, China) in the TNBS + EA group. For the sham EA, at the same treatment points, the TNBS group received electrodes without needle penetration into the skin and current was set to 0 while the control group only received topical disinfectant. All treatments were conducted for 30 min daily for consecutive 14 days. The body weight and average food intake were recorded and calculated daily for each rat. At the end of the experiment, behavioral tests were conducted and the feces were collected. The experimental procedures were shown in Figure 1(a). Hypothalamic pituitary tissues were gathered and placed in liquid nitrogen for rapid freezing. The blood samples were acquired and centrifuged (13000 RPM, 4°C, 10 min) to isolate the supernatant. Then, these samples were stored at −80°C refrigerator for ELISA analysis. The hippocampal tissues were isolated. Parts of the tissues were fixed in 4% paraformaldehyde for immunofluorescence staining, and the rest were stored in −80°C for ^1^H NMR spectroscopy.

All animal experiments were conducted in accordance with the Institutional Animal Care guidelines approved by the Experimental Animal Ethical Committee of the Zhejiang Chinese Medical University (no. ZSLL-2018-014).

### 2.2. Behavioral Testing

The elevated plus maze (EPM) and open-field test (OFT) are commonly used in rodents to assess the exploratory and locomotor activity, unconditioned fear, and potential anxiety [31]. The EPM consists of open-arms and closed-arms that extend crosswise from the central platform. The OFT is composed of a black wall and base (the bottom surface is divided equally into 16 squares). Rats were allowed to adjust to the environment for 5 minutes. A video camera was suspended above and captured the movement of each rat for 5 min. The total number of entries, the proportion of open-arms entries [(open − arms entries/total arms entries) × 100%] and time spent in open-arms [(open − arms time/total arms time) × 100%] of EPM, the total movement distance, the percentage of distance spent in the central region, and the time spent in central areas of OPT were calculated by the SMART 3.0 system (Panlab, Barcelona, Spain).

### 2.3. 16S rDNA Sequencing and Bioinformatic Analysis

Microbial total genomic DNA of each fecal sample (200 mg) was extracted using a DNA extraction kit (E. Z. N. A. ®Stool DNA Kit, D4015, Omega, Inc., USA). The V3-V4 regions of the bacterial 16S rDNA gene were amplified with primers 341F (Forward primer: 5′-CCTACGGGNGGCWGCAG-3′) and 805R (Reverse primer: 5′-GACTACHVGGGTATCTAATCC-3′). The operation was performed as described elsewhere [32]. The feature sequences were obtained from the DADA2 core denoising function of QIIME 2 and used with the Amplicon Sequence Variants (ASVs) to construct the equivalent of 100% clustering operational classification units (OTUs). Alpha diversity and beta diversity were calculated by QIIME 2 after being normalized by the SILVA classifier (release 132). Feature sequence alignment was measured with Blast. Annotation and analysis were performed with SILVA and NT-16S databases. Graphs were generated with the R vegan package (version 3.5.2).

### 2.4. Sample Preparation for Metabolic Profiling

The thawed samples of the hippocampus (80 mg) and feces (100 mg) were dissolved with 800 *μ*L of mixture of PBS with 10% deuterated water (99.8% D2O) containing 0.005% sodium 3-trimethylsilyl-propionate-d4 (TSP, SIGMA, USA), which was used as the chemical shift reference. Samples were homogenized by Tissue Lyser II (Retsch, Germany) at a frequency of 1/60 for 2 min and then centrifuged at 13000 RPM for 10 min (4°C). A volume of 500 *μ*L of the clear supernatant was transferred to 5 mm NMR tubes for ^1^H NMR measurements.

### 2.5. ^1^H NMR Spectroscopy Analysis and Data Processing

^1^H NMR was analyzed by a Bruker 600 MHz AVANCE III spectrometer equipped with a 5 mm-BBFO probe (ambient temperature 24°C). The manipulation was performed according to the previous study [24]. The defining peaks of the sample metabolites were analyzed in accordance with previous publications [33–36] and the network database of metabolomics, including the Biological Magnetic Resonance Bank (http://www.bmrb.wisc.edu/metabolomics) and the Human Metabolome Database (http://www.hmdb.ca/). The partial least squares discriminant analysis (PLS-DA) was performed using SIMCA 14.2 (Umetrics AB, Umea, Sweden) after metabolic data were normalized to constants and scaled.

### 2.6. Immunofluorescence Staining and Image Analysis

Hippocampus tissue was fixed with 4% paraformaldehyde for 24 h (room temperature). Sections were embedded and cut into slices, dewaxed, and soaked in antigen retrieval solutions (Citric Acid, PH6.0, G1202). Blocking was done using Bovine Serum Albumin (BSA, G5001) for 30 min and incubation at 4°C overnight with the specific primary antibodies. Then, samples were washed with PBS for 3 × 5 min and incubated with the corresponding secondary antibody (Table 1). The autofluorescence quenching agent was added, and the nucleus was restained with DAPI (G1012) and covered with antifade fluorescent mounting medium (G1401). Ultimately, the immunofluorescence results were observed with a fluorescence microscope (Nikon Eclipse C1, NIKON, Japan). The images were calculated with Image-Pro Plus 6.0 (Media Cybemetics, U.S.A). The average optical density is equal to integrated optical density divided by the area for each representative picture (AOD = IOD/Area) and was used as variables for statistical analysis (three observed fields for each slice). All the chemical reagents were purchased from Servicebio Technology and Biotechnology Co., Ltd., China.

### 2.7. ELISA

The tissues of hypothalamic pituitary were thawed and homogenized in PBS solution, centrifuged at 13000 RPM, 4°C, for 10 min, and the supernatants were isolated. Serum was thawed and prepared. Then, ELISA was conducted according to the manufacturer's protocol. The concentration of corticotropin-releasing hormone (CRH) in the hypothalamus, the adrenocorticotropic hormone (ACTH) in pituitary gland, and the contents of CRH, ACTH, and CORT in serum were measured by ELISA assay kit (Fankew, Shanghai Fankel Industrial Co., Ltd).

### 2.8. Statistical Analysis

Data analyses were conducted with GraphPad Prism 9.0. Differences between two groups were applied using an independent sample *t* test. While among three groups, a one-way analysis of variance (ANOVA) followed by Bonferroni's post hoc test was assessed. All values were expressed as mean ± standard deviation (SD). The alpha diversity and beta diversity, the principal components analysis (PCA), and the principal coordinate analysis (PCoA) were tested using Wilcoxon's rank-sum test. The relative abundance of bacteria was calculated (as X bacterial count/total count) by Kruskal-Wallis test. In all cases, the value of *P* < 0.05 was defined as statistical significance.

## 3. Results

### 3.1. EA at ST36 and SP6 Alleviated Behavioral Symptoms

The average daily food intake, body weight, and colon length of rats in the TNBS group were significantly lower than that in the control group (average food intake: *P* < 0.001; days 2-14 of body weight: *P* < 0.05; colon length: *P* < 0.001) but were reversed by EA treatment (average food intake: *P* < 0.05; days 2-7 of body weight: *P* < 0.05; colon length: *P* < 0.01) (Figures 1(b)–1(e)).

**Figure 2:**
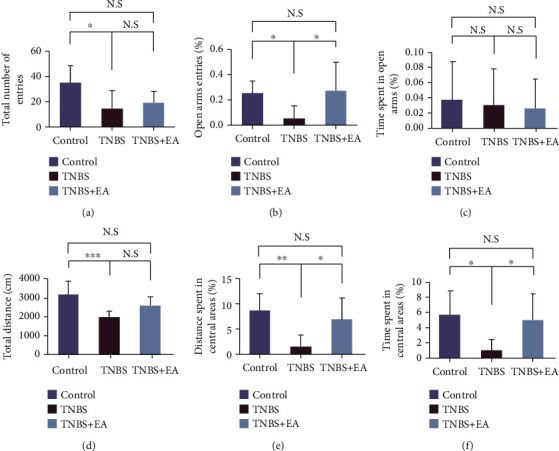
EA at ST36 and SP6 alleviated behavioral symptoms (control (*n* = 8), TNBS (*n* = 7), and TNBS + EA group (*n* = 7)). (a)–(c) EPM: the total number of entries, the open-arms entries, and the percentage of time spent in open-arms. (d)–(f) OFT: the total distance, the percentage of distance, and time spent in central areas. Data were shown as mean ± SD. ∗*P* < 0.05, ∗∗*P* < 0.01, ∗∗∗*P* < 0.001 by Bonferroni's post hoc test.

To assess TNBS-induced anxiety and depression-like behaviors in rats and evaluate the corresponding therapeutic effects of EA, we carried out behavioral tests. In the EPM test (Figures 2(a)–2(c)), the total number of entries and percentage of entries into open-arms areas was significantly lower when treated with TNBS compared to the control. However, the proportion of entries into open-arms was significantly increased after EA treatment (*P* < 0.05). The proportion of time spent in open-arms shows no differences among the three groups. From the OFT test (Figures 2(d)–2(f)), the behavioral characteristics of the TNBS group were significantly lower compared to the control group (*P* < 0.05). And the percentage of distance and time spent in central areas was significantly increased by EA therapy (*P* < 0.05). The results suggested that EA could improve TNBS-induced anxiety and depression-like behaviors.

### 3.2. EA at ST36 and SP6 Changed the Gut Microbiome Composition

**Figure 3:**
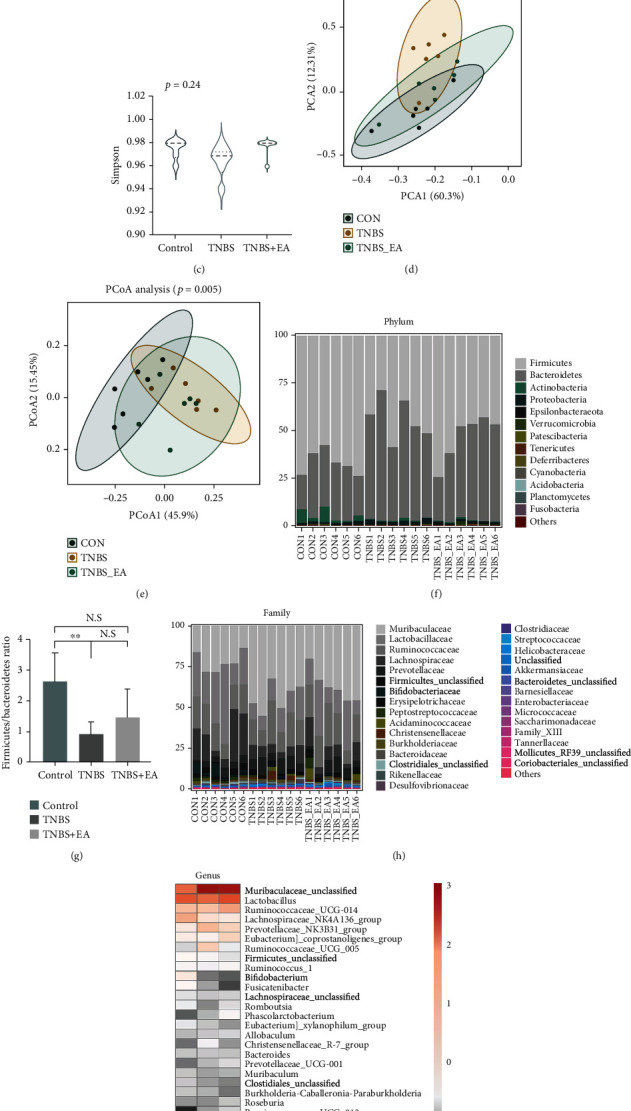
EA at ST36 and SP6 altered the gut microbiota community composition (*n* = 6/group). (a) Chao1 index analysis. (b) Shannon index analysis. (c) Simpson index analysis. (d) and (e) PCA and PCoA scatter plot analysis. (f) Microbiota compositions in phylum level. (g) The ratio of Firmicutes/Bacteroidetes. Data were shown as mean ± SD. ∗∗*P* < 0.01 by Bonferroni's post hoc test. (h) Microbiota compositions in the family level. (i) Microbiota compositions in genera level.

The alpha diversity index reflected the richness and evenness of species. As showed in Figures 3(a)–3(c), we observed that Chao1, Shannon, and Simpson indexes were the lowest in the TNBS group. But there was no statistical difference among the three groups. Beta diversity including the PCA and PCoA scatter plot represented the differences in microbiota between samples. Specifically, it showed the closer the distance, the smaller the difference in the intestinal microbiota; on the contrary, the farther the distance, the greater the difference. Obviously, the distance of the TNBS + EA group was closer to the control group but away from the TNBS group (Figures 3(d) and 3(e); *P* < 0.01).

The alterations in microbial composition at the level of the top 13 phyla, 30 families, and 30 genera were analyzed based on the observed microbial species of 17 phyla, 147 families, and 313 genera, with the abundance in total coverage of approximately 99%, 99%, and 94%, respectively. The details were listed in Table 2. At the phyla level (Figure 3(f)), the relative abundance of *Firmicutes* was lower and *Bacteroidetes* was higher in the TNBS group compared to the control group, but both were reversed by EA treatment. The ratio of *Firmicutes/Bacteroidetes* (F/B) was raised by EA (Figure 3(g)), which was reported as a key index for a healthy state of the gut microbiome [37], and positively correlated with body weight [38]. Furthermore, after EA therapy, an increase in the relative abundance of *Verrucomicrobia* was also observed alongside a decrease in the level of *Actinobacteria*.

At the family level (Figure 3(h)), EA increased the reduction of the relative abundance of *Lactobacillaceae* and *Acidaminococcaceae* in TNBS in comparison to the control group with a significantly decreased abundance of *Muribaculaceae*. After EA treatment, the relative abundance of *Akkermansiaceae* exhibited a further increase (*P* < 0.05), which is beneficial to mucosal development [39]. Nevertheless, the relative abundance of *Bifidobacteriaceae* was decreased. The genus level was visualized with a heat map (Figure 3(i)). EA increased the reduction of the relative abundance of *Lactobacillus*, *Ruminococcaceae_UCG-014*, *and Romboutsia* in TNBS relative to the control group, but decreased the relative abundance of *Muribaculaceae_unclassified* (*P* < 0.05) and *Alistipes* (opportunistic pathogen). In addition, the level of *Phascolarctobacterium* and *Blautia* was increased.

To summarize, these results indicated that EA may treat TNBS-induced colitis in rats by readjusting the gut microbiome.

### 3.3. EA at ST36 and SP6 Altered Metabolism in the Hippocampus

**Figure 4:**
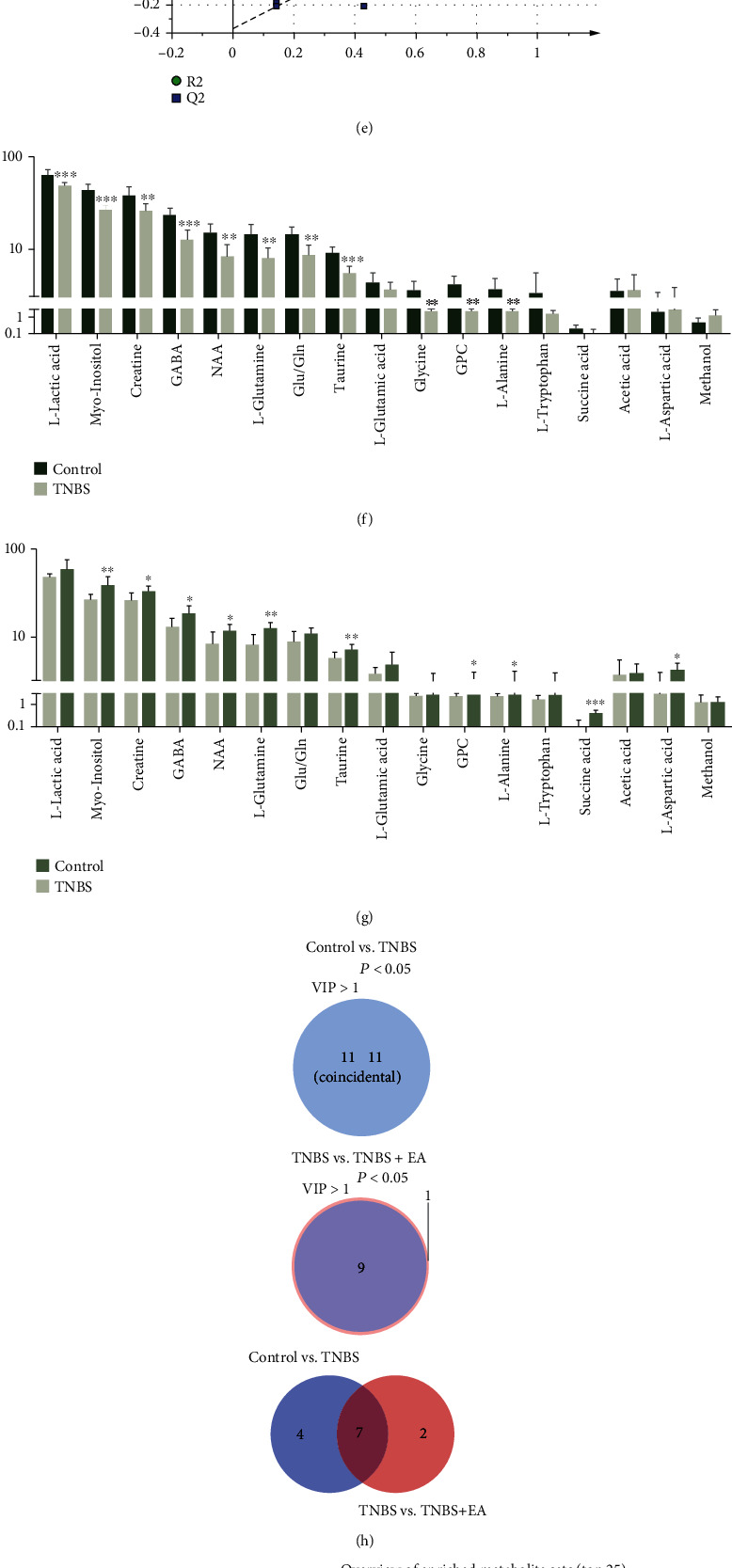
EA at ST36 and SP6 altered metabolism in the hippocampus (*n* = 7/group). (a)–(c) PLS-DA scatter plot results of metabolites. (d) and (e) Validation model plot. (f) and (g) Differential metabolites between the control and TNBS group, and the TNBS and TNBS + EA group, respectively. Data were shown as mean ± SD, ∗*P* < 0.05, ∗∗*P* < 0.01, ∗∗∗*P* < 0.001 by the independent sample *t* test. (h) Venn diagrams demonstrate the number of altered metabolites between the control and TNBS group, the TNBS and TNBS + EA group, and the control vs. TNBS (blue) and TNBS vs. TNBS + EA group (pink). (i) The differential of meaningful metabolic pathways among the control, TNBS, and TNBS + EA group.

^1^H NMR spectroscopy detected that there are 17 neural metabolites that were altered in the hippocampus, including l-lactic acid, myo-inositol, creatine, gamma-aminobutyric acid (GABA), *N*-acetyl-_L_-aspartic acid (NAA), l-glutamine, glutamate/glutamine (Glu/Gln), taurine, l-glutamic acid, glycine, glycerophosphocholine (GPC), l-alanine, l-tryptophan, succinic acid, acetic acid, l-aspartic acid, and methanol. The PLS-DA scatter showed that TNBS + EA was closer to the control but farther from the TNBS group (Figures 4(a)–4(c)), which indicated that the composition of metabolites in TNBS + EA group has more similarity with the control group. Moreover, the model of the PLS-DA was stable and reliable, with parameters as follows: control vs. TNBS: *R*2*X* = 0.552, *R*2*Y* = 0.989, *Q*2 = 0.954; TNBS vs. TNBS + EA: *R*2*X* = 0.474, *R*2*Y* = 0.890, *Q*2 = 0.741 (Figures 4(d) and 4(e)).

Compared with the control group, except for the acetic acid, l-aspartic acid and methanol were increased, and the other 14 metabolites were decreased in the TNBS group (Figure 4(f)). Nevertheless, 10 metabolites containing myo-inositol, creatine, GABA, NAA, l-glutamine, taurine, GPC, l-alanine, succinic acid, and l-aspartic acid were significantly upgraded by EA treatment (*P* < 0.05), and the *P* value of Glu/Gln is 0.064 (Figure 4(g)). Furthermore, based on the criteria of multivariate statistical analysis of both influence on projection (VIP) > 1 and *P* < 0.05 were used to identify significant changes in metabolites intragroup. In Venn diagrams (Figure 4(h)), a total of 11 (VIP > 1) or 11 (*P* < 0.05) metabolites were determined to be unique between the control and TNBS group. All were selected with the double criteria. In addition, 9 (VIP > 1) or 10 (*P* < 0.05) differential metabolites were determined between TNBS group and TNBS + EA group, and 9 of them were selected with the double criteria. Taken together, there were 7 overlapping metabolites, including GABA, NAA, creatine, GPC, l-glutamine, myo-Inositol, and taurine between the control vs. TNBS and TNBS vs. TNBS + EA group. Then, we identified the corresponding enrichment pathways for the differential metabolites through the MetaboAnalyst 5.0 website (https://www.metaboanalyst.ca/) with the condition of an impact value > 0.1, *P* < 0.05, and false discovery rate (FDR) *q* value < 0.1. Accordingly, the alanine, aspartate, and glutamate metabolism pathway showed a significant difference among the three groups (Figure 4(i)). The results implied that the antianxiety and antidepressive effects of EA may be through a reversal of the amino acid metabolism pathway.

### 3.4. EA at ST36 and SP6 Blocked the Activation of TLR4/Nuclear Factor-Kappa B (NF-*κ*B) Signaling Pathway and NLRP3 Inflammasomes

**Figure 5:**
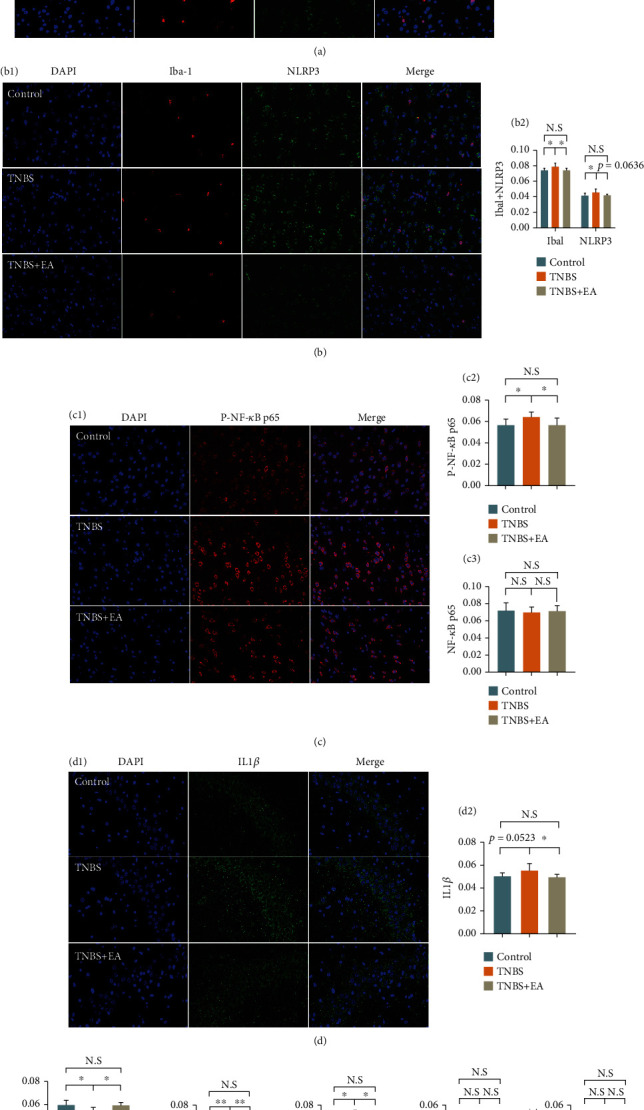
EA at ST36 and SP6 blocked the activation of TLR4/NF-*κ*B signaling pathway and NLRP3 inflammasomes (*n* = 3/group). Representative images of immunofluorescence staining of hippocampal slices for DAPI (blue, nuclei indicator), Iba − 1 (red) + TLR4 (green), Iba − 1 (red) + NLRP3 (green), P-NF-*κ*B p65 (red), and IL-1*β* (green). Original magnification ×400. (a1) and (a2) The immunofluorescence staining and histogram of Iba1 + TLR4. (b1) and (b2) The immunofluorescence staining and histogram of Iba1 + NLRP3. (c1)–(c3) The immunofluorescence staining and histogram of P-NF-*κ*B p65 and the histogram of NF-*κ*B p65. (d1) and (d2) The immunofluorescence staining and histogram of IL-1*β*. (e) The histogram of ZO-1. (f) The histogram of MyD88. (g) The histogram of TAK1. (h) The histogram of p38 MAPK. (i) The histogram of P-p38 MAPK. Data were shown as mean ± SD. ∗*P* < 0.05 and ∗∗*P* < 0.01 by Bonferroni's post hoc test.

To verify whether EA treatment modulates this molecular signaling mechanism, we detected and quantified the TLR-4/NF-*κ*B and MAPK and NLRP3 inflammasomes, which is a risk factor of depression [40, 41]. Immunofluorescence staining was shown in Figure 5. The indicators except IL-1*β* (*P* = 0.0523) were significantly higher in the TNBS group compared to the control group (*P* < 0.05). However, EA markedly downregulated (*P* < 0.05) the expressions of Iba-1 (microglia activation marker) (Figures 5(a1)–5(b2)), TLR4 (Figures 5(a1) and 5(a2)), myeloid differentiation primary response 88 (MyD88) (Figure 5(f)), transforming growth factor-*β*-activated kinase 1 (TAK1) (Figure 5(g)), P-NF-*κ*B p65 (Figures 5(c1) and 5(c2)), and interleukin- (IL-) 1*β* (Figures 5(d1) and 5(d2)) and improved the expressions of NLRP3 (*P* = 0.0636; Figures 5(b1) and 5(b2)). There was no statistical difference in the levels of NF-*κ*B p65; actually, P-NF-*κ*B p65 is an essential downstream event upon TLR4 activation.

Unexpectedly, neither the p38 mitogen-activated protein kinase (MAPK) nor the P-p38 MAPK generated a noticeable difference among the three groups (Figures 5(h) and 5(i)), suggesting that EA may not affect their expression in the hippocampus. These results indicated that TNBS induced the activation of the TLR4/NF-*κ*B signaling pathway and NLRP3 inflammasomes and then increased the expression of IL-1*β*. Whereas the inflammatory cytokines were decreased by EA administration. Furthermore, we detected the expression of the tight junction protein of ZO-1 and found that EA prevented the decrease of ZO-1 in TNBS-induced rats (*P* < 0.05, Figure 5(e)). Thus, EA may have improved permeability of the blood brain barrier (BBB). The representative images of immunofluorescence staining of NF-*κ*B p65, ZO-1, MyD88, TAK1, p38 MAPK, and P-p38 MAPK were shown in supplementary materials (available here).

### 3.5. EA at ST36 and SP6 Downregulated the HPA Axis Activity

**Figure 6:**
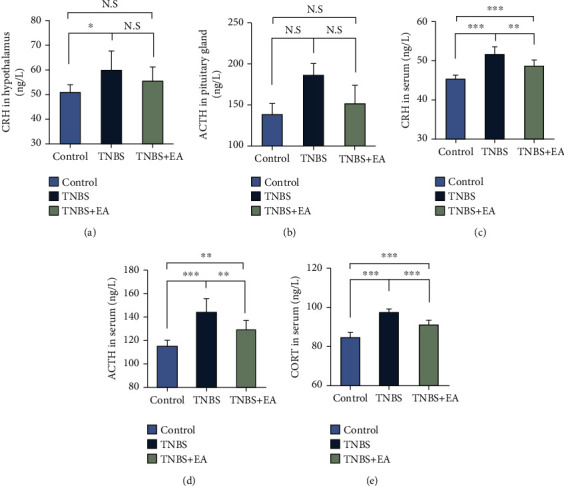
EA at ST36 and SP6 downregulated the HPA axis activity (control (*n* = 8), TNBS (*n* = 7), and TNBS + EA group (*n* = 8)). (a) The concentrations of CRH in hypothalamus. (b) The concentrations of ACTH in pituitary gland. (c)–(e) The concentrations of CRH, ACTH, and CORT in serum. Data were shown as mean ± SD. ∗*P* < 0.05, ∗∗*P* < 0.01, ∗∗∗*P* < 0.001 by Bonferroni's post hoc test.

Our study found that the concentrations of CRH in the hypothalamus and the CRH, ACTH, and CORT in the serum were significantly higher in the TNBS group compared with the control group (*P* < 0.05). CRH, ACTH, and CORT in serum were significantly decreased by EA treatment (*P* < 0.05) (Figures 6(a)–6(e)). However, the concentrations of CRH in the hypothalamus and ACTH in the pituitary gland showed no significant difference between TNBS and TNBS + EA group, which may be modulated by the negative feedback mechanism. In summary, EA dramatically attenuated the hyperactivity of the HPA axis.

## 4. Discussion

The colon length and the average food intake of rats in the TNBS group were significantly decreased compared with the control group, but increased by EA treatment. However, there was no significant weight gain from day 8 onwards. On the one hand, TNBS may cause intestinal flora dysbiosis and severely impaired intestinal function, which affects digestion and absorption. On the other hand, Turnbaugh et al. proposed that the rise in the relative abundance of Firmicutes was associated with increased energy storage, leading to obesity, while *Bacteroidetes* were correlated with weight loss [42]. Interestingly, the ratio of F/B in the TNBS group was the lowest among these groups, with an improved EA. This indicates that the benefits of EA in preventing weight loss during treatment may act through upregulation of the F/B ratio.

The primary source of short chain fatty acids (SCFAs) is the fermentation of indigestible dietary, which influences metabolism and mucosal immunity, maintains gut epithelial integrity, influences neuropsychiatric disorders, and improves psychological functioning, and SCFAs are therefore speculated to be mediators in the crosstalk of the MGB axis [43]. As expected, the relative abundances of SCFAs-producing bacteria, including *Ruminococcaceae*, *Phascolarctobacterium*, *Akkermansiaceae*, *Romboutsia*, and *Blautia* were increased by EA therapy. This may serve as evidence that EA improves anxiety and depression-like behaviors through the MGB axis mediated by SCFAs.

Moreover, the relative abundance of *Lactobacillaceae* was increased after EA treatment and was able to effectively alleviate intestinal inflammation [44]. Unexpectedly, our present study found that there was no improvement of the *Bifidobacteriaceae* levels and a decreased the relative abundance of *Muribaculaceae* (previously known as *S24-7*). The variation of microbial diversity and composition may be affected by integrated mechanisms such as individual host differences, the microbial microenvironment, and the dominant gut microbiota interaction with the host.

Previous studies have found disturbances in glutamate and lipid metabolism in the hippocampus of depressed rat models, which may reflect the function of these pathways involved in the development of depression [45]. Interestingly, the study concluded that EA markedly improved the disturbance of amino acid metabolism in the hippocampus. Glutamate is found in more than 80% of all neurons, and the glutamatergic systems play a vital role in the pathogenesis of depression [46]. GABA is the key inhibitory neurotransmitter in adult mammals, which is directly involved in anxiety and depression. Briefly, various antidepressant manipulations have been shown to increase the functional decline of GABAergic neurons, thereby improving anxiety and depressive symptoms [47, 48]. The negative behavior may be stimulated by interfering with the glutamate-glutamine-GABA cycle (Gln-Glu-GABA cycle) in the hippocampus, cerebellum, and striatum region [49]. Consistent with the above, the levels of GABA, l-glutamine, and Glu/Gln were significantly decreased in the TNBS group compared to the control group, but all of these metabolites were reversed by EA treatment. Therefore, EA may regulate the synthesis of glutamate and the production of GABA, which then contributes to the generation of cooperative action in brain regions via the Gln-Glu-GABA cycle.

EA also corrected the disturbance of myo-inositol, creatine, NAA, taurine, GPC, l-alanine, succinic acid, and l-aspartic acid. Creatine functions in the adenosine triphosphate cycle [50]. Taurine can regulate lipid metabolism and serve as an antioxidant [51]. Succinic acid is an intermediate product of the tricarboxylic acid cycle and supports the stability of gut microbiota [52]. As a result, we hypothesized that the antianxiety and depression-like effects of EA were primarily mediated through the amino acid metabolic pathway with the synergistic effects of multiple metabolites.

Microglia are considered as mediators of central immune signal transduction in psychological illness through the activation of TLR4-dependent signaling and the NLRP3 inflammasome pathway [40, 53, 54]. TLR4 stimulates the MyD88 dependent signaling pathway. This pathway signals the activation of TAK1 and phosphorylation of NF-*κ*B p65. The TLR4/NF-*κ*B signal pathway relieves the autoinhibition of NLRP3, which functions through the adaptor protein ASC to recruit pro-caspase-1 through its caspase recruitment domain. This then assembles together to form the NLRP3 inflammasome and shears pro-IL-1*β* (and pro-IL-18) into mature IL-1*β* (and IL-18) [55]. Consistently, our study found that IL-1*β* expression as well as anxiety and depression-like behavior of rats were increased in TNBS group compared to the control group, but reversed by EA treatment. Besides, the gut microbiome releases molecular metabolites that can increase the intestinal blood barrier and the permeability of the BBB [56]. The immunofluorescence staining found that the expression of ZO-1, a biomarker of permeability, showed consistent alterations with IL-1*β*. Thus, enhanced epithelial barrier function was necessary to anti-inflammatory cascades.

Although the NLRP3 has been the most studied inflammasome in the CNS, it is still controversial at present. Burm et al. reported that the NLRP3 may not be necessary, because the transformation of the inflammasome into a mature form of IL-1*β* in innate immune cells in the CNS is not strictly dependent on caspase-1 [57]. However, based on the above findings, we tend to speculate that EA enhanced BBB function and inhibited TLR4/NF-*κ*B signaling pathway and NLRP3 inflammasome. Thereby the sequence improved inflammation and behavioral tests in TNBS-induced IBD rats.

Furthermore, previous studies suggested that activation of microglia, including activation of released NLRP3 inflammasome and IL-1*β*, may be induced by CORT [41, 54]. And the inflammatory cytokines directly stimulate HPA axis and indirectly alter the sensitivity of GRs to cortisol. Although GRs are widely present in brain regions and peripheral organs, the hippocampus has a high density of GRs and is thereby highly susceptible to CORT concentrations. Once released from the hypothalamus, CRH signals the release of ACTH from the anterior pituitary during the stress response. ACTH then signals for the release of corticosterone in rodents (cortisol in humans) [29, 31]. Many effects of CORT on the glutamate system are achieved by binding to GRs in the cell membrane and then further translocation to the nucleus and regulation of transcription of target genes. GABA controls epigenetic and gene transcription responses to psychological stress by suppressing the secretion of CORT [21, 58]. Taken together, our results suggest that EA may be more beneficial to the improvement of inflammation and metabolic disorders in the hippocampal region by alleviating the overactivity of the HPA axis.

There are some limitations within our study. First, the experiment is performed by inserting needles for each administration, which may induce pain in rats and generate unwanted stress effects [59]. Second, the analyses about proteins expression by immunofluorescence were limited to the observed field of view. We have not further validated methods as western blot or RT-PCR in this study. Third, many intermediate or low concentration metabolites may not be well represented in ^1^H NMR, which may result in biases inherent in incomplete annotations. Thus, other techniques (e.g., liquid chromatography-tandem mass spectrometry and gas chromatography-mass spectrometry) may be necessary.

## 5. Conclusions

**Figure 7:**
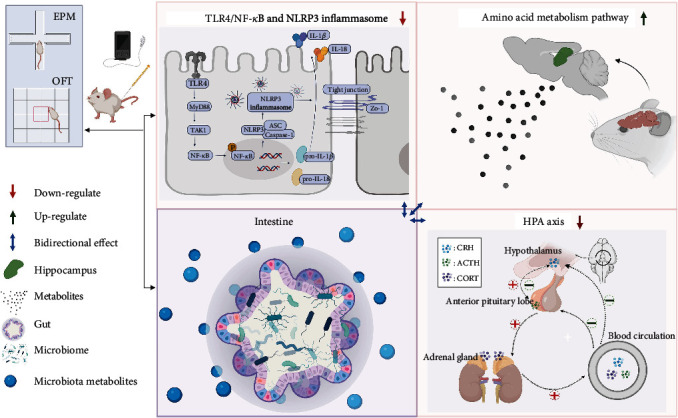
Summary and speculation of this study on the potential mechanisms of EA treatment for TNBS-induced anxiety and depression-like behaviors in rats. Bidirectional relationship between the gut microbiome and the brain.

In summary, our study concluded that EA at ST36 and SP6 improved the anxiety and depressive symptoms in rats, which may be caused by the suppression of the inflammatory response of the hippocampal microglia and the overactivity of the HPA axis. Besides, EA also enhanced the BBB function and amino acid metabolism so the composition of the gut microbiota can be modulated via the MGB axis. The brain and gut microbiota likely underwent a complex interaction of bidirectional regulatory mechanisms, ultimately alleviating anxiety and depression-like behavior in TNBS-induced colitis rats (Figure 7). Though EA is a nontraditional antidepressant treatment, our study provides evidence for the clinical application of EA as an adjunctive treatment for IBD-related antianxiety and depression.

## Figures and Tables

**Table 1:** Antibodies used in immunofluorescence staining.

Antibody	Dilution
Primary antibody	
Rabbit anti-Zo1 (GB111402)	1 : 500
Mouse anti − Iba1 + rabbit anti − TLR4 (GB12105 + GB11519)	1 : 300 + 1 : 1000
Mouse anti − Iba1 + rabbit anti − NLRP3 (GB12105 + GB11519)	1 : 300 + 1 : 1000
Rabbit anti-MyD88 (GB111402)	1 : 200
Rabbit anti-TAK1 (GB11701)	1 : 300
Rabbit anti-NF-*κ*B p65 (GB111053)	1 : 100
Rabbit anti-P-NF-*κ*B p65 (GB13025-1)	1 : 100
Rabbit anti-p38 MAPK (GB13448)	1 : 300
Rabbit anti-P-p38 MAPK (GB13006-1)	1 : 100
Rabbit anti-IL-1*β* (GB11113)	1 : 200
Secondary antibody	
Alexa fluor 488 goat anti-rabbit (GB25303)	1 : 300~1 : 400
Alexa fluor CY3 goat anti-mouse (GB25301)	1 : 300

**Table 2:** The alterations in microbial composition at the level of the top 13 phyla, 30 families, and 30 genera of the TNBS + EA group compared to the TNBS group.

Level	Regulation by EA			
	Increased (X1 bacteria)	Decreased (X2 bacteria)	Further increased (X3 bacteria)	Further decreased (X4 bacteria)
Phylum	*Firmicutes*∗ *Patescibacteria* *Tenericutes* *Planctomycetes*	*Bacteroidetes*∗ *Proteobacteria*∗ *Deferribacteres* *Cyanobacteria*∗ *Fusobacteria*	*Epsilonbacteraeota* *Verrucomicrobia*∗ *Acidobacteria*	*Actinobacteria*∗

Family	*Lactobacillaceae* *Lachnospiraceae* *Prevotellaceae* *Erysipelotrichaceae* *Peptostreptococcaceae*∗ *Acidaminococcaceae*∗ *Saccharimonadaceae* *Family_XIII* *Mollicutes_RF39_unclassified*	*Muribaculaceae*∗ *Christensenellaceae* *Burkholderiaceae* *Bacteroidaceae* *Rikenellaceae* *Desulfovibrionaceae* *Bacteroidetes_unclassified* *Enterobacteriaceae*∗ *Micrococcaceae* *Tannerellaceae* *Coriobacteriales_unclassified*	*Ruminococcaceae* *Clostridiaceae* *Streptococcaceae* *Helicobacteraceae* *Unclassified* *Akkermansiaceae*∗	*Firmicutes_unclassified* *Bifidobacteriaceae*∗ *Clostridiales_unclassified* *Barnesiellaceae*

Genus	*Lactobacillus* *Ruminococcaceae_UCG-014* *Eubacterium]_coprostanoligenes_group* *Ruminococcus_1* *Lachnospiraceae_unclassified* *Romboutsia*∗ *Muribaculum*	*Muribaculaceae_unclassified*∗ *Prevotellaceae_NK3B31_group* *Ruminococcaceae_UCG-005* *Christensenellaceae_R-7_group* *Bacteroides* *Alistipes*	*Phascolarctobacterium*∗ *Allobaculum* *Roseburia* *Ruminococcaceae_UCG-013* *Streptococcus* *Helicobacter* *Blautia*∗ *Unclassified*	*Lachnospiraceae_NK4A136_group* *Firmicutes_unclassified* *Bifidobacterium*∗ *Fusicatenibacter* *Eubacterium]_xylanophilum_group* *Prevotellaceae_UCG-001* *Clostridiales_unclassified* *Burkholderia-Caballeronia-Paraburkholderia* *Intestinimonas*

Notes: With regard to the TNBS group, the concentration of X1 bacteria decreased while the concentration of X2 bacteria increased compared with the control group. EA treatment was able to restore the natural balance of these organisms (X1 bacteria increased; X2 bacteria decreased). With regard to the TNBS group, the concentration of X3 bacteria increased while the concentration of X4 bacteria decreased when compared to the control group. EA treatment exacerbated these changes by further elevating the concentration of X3 bacteria and lowering the concentration of X4 bacteria. (∗*P* < 0.05 by Kruskal-Wallis test).

## Data Availability

All data and results needed to support the conclusions of this paper are in the paper and/or supplementary materials, for additional information, please contact the authors.

## Supplementary Materials

Supplementary Materials We have provided supplementary material in a separate file. The representative images of immunofluorescence staining of NF-*κ*B p65, ZO-1, MyD88, TAK1, p38 MAPK, and P-p38 MAPK.
