# Supplementary material for: Electroacupuncture Attenuated Anxiety and Depression-Like Behavior via Inhibition of Hippocampal Inflammatory Response and Metabolic Disorders in TNBS-Induced IBD Rats
Source: Oxid Med Cell Longev. 2022 Jan 18;2022:8295580. doi: 10.1155/2022/8295580 (PMC8789424; doi:10.1155/2022/8295580)
Supplement: Supplementary Materials — We have provided supplementary material in a separate file. The representative images of immunofluorescence staining of NF-κB p65, ZO-1, MyD88, TAK1, p38 MAPK, and P-p38 MAPK. [file 8295580.f1.pdf]

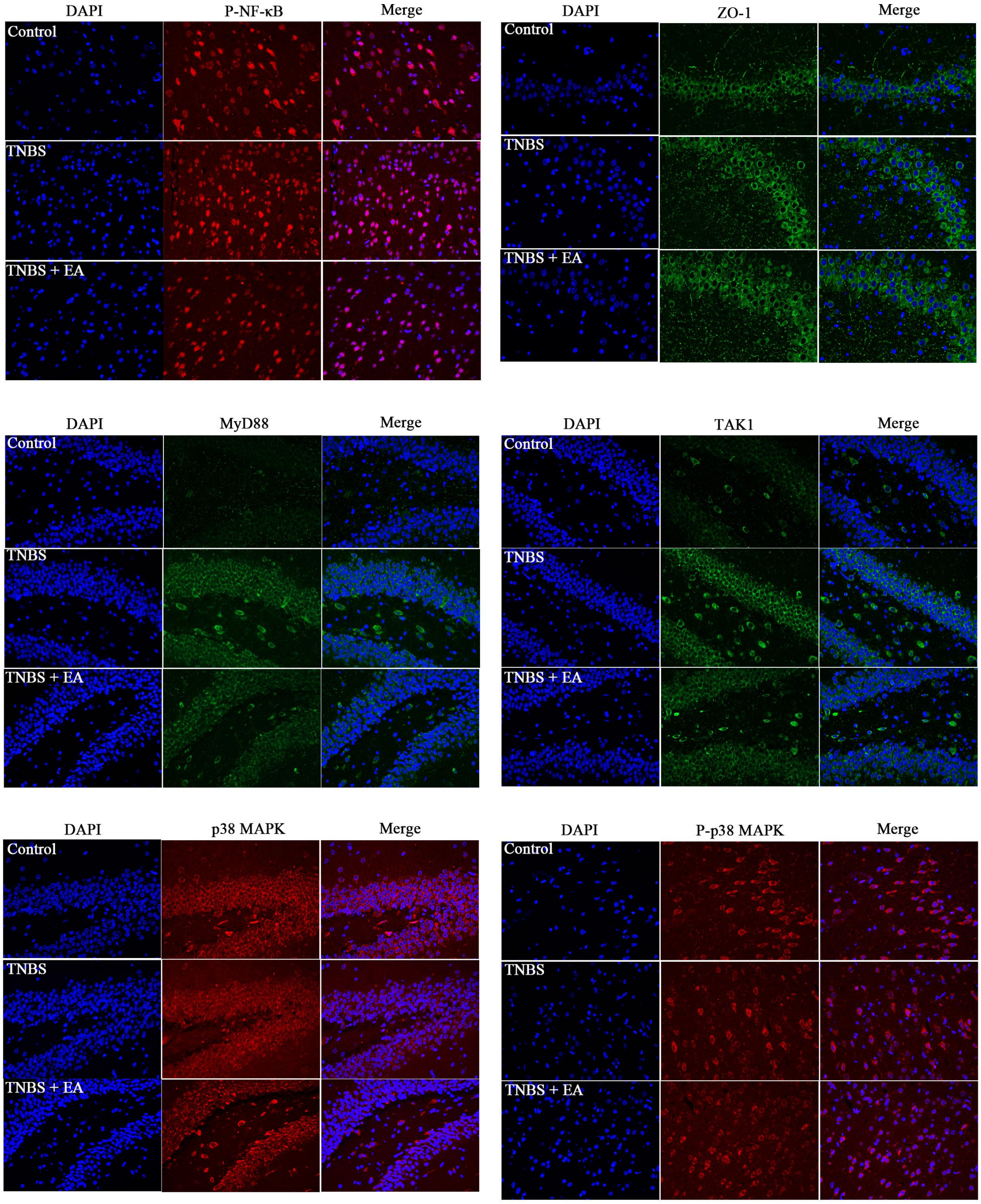

Supplementary material. The representative images of immunofluorescence staining of NF-κB p65 (red) , ZO-1 (green), MyD88 (green), TAK1 (green), p38 MAPK (red) and P-p38 MAPK (red) . Original magnification × 400.
